# Feedback beyond accuracy: Using eye‐tracking to detect comprehensibility and interest during reading

**DOI:** 10.1002/asi.24657

**Published:** 2022-05-24

**Authors:** Frans van der Sluis, Egon L. van den Broek

**Affiliations:** ^1^ Department of Communication University of Copenhagen Copenhagen Denmark; ^2^ Department of Information and Computing Sciences Utrecht University Utrecht the Netherlands

## Abstract

Knowing what information a user wants is a paramount challenge to information science and technology. Implicit feedback is key to solving this challenge, as it allows information systems to learn about a user's needs and preferences. The available feedback, however, tends to be limited and its interpretation shows to be difficult. To tackle this challenge, we present a user study that explores whether tracking the eyes can unpack part of the complexity inherent to relevance and relevance decisions. The eye behavior of 30 participants reading 18 news articles was compared with their subjectively appraised comprehensibility and interest at a discourse level. Using linear regression models, the eye‐tracking signal explained 49.93% (comprehensibility) and 30.41% (interest) of variance (*p* < .001). We conclude that eye behavior provides implicit feedback beyond accuracy that enables new forms of adaptation and interaction support for personalized information systems.

## 1 INTRODUCTION

Understanding and knowing what the user wants in terms of information is a paramount challenge for information systems (Saracevic, 2007). Text mining techniques are currently employed to infer a user's information need through estimating whether a document is similar to a query (information retrieval), is popular among similar people or friends (collaborative filtering), or is similar to a user model (cognitive filtering). Even though these techniques are unmistakably successful, they seem to be plagued by a magic barrier: A limit in their potential to predict the value of information (Said & Bellogín, 2018; Voorhees, 2002). Current techniques struggle to adapt to differences between users, such as their knowledge and preferences, as well as to differences within users, such as changing information needs and interests (Hill et al., 1995). Because the value of information differs per user and changes over time (Belkin, 2008; Saracevic, 2007), continuous feedback is needed to better predict whether and when information is valuable to a user (Ghorab et al., 2013; Liu et al., 2020).

Asking users to provide copious and continuous input about the information they want is not likely to succeed. Instead, implicit feedback that does not require any interaction from the user is a more viable option (Barral et al., 2016; Ghorab et al., 2013; Liu et al., 2020). Basic on‐line measures have already been successfully leveraged for text mining. Features from click‐stream data, browsing data, and query‐text relations enhance binary ranking precision by up to 31% (Agichtein et al., 2006) and predict graded relevance assessments by up to *r* = .411 (Guo & Agichtein, 2012). Additionally, physiological signals (Barral et al., 2016) and, in particular, eye‐tracking (Li et al., 2018) holds the promise to expand on these results (Cole et al., 2015). Human attention follows a distinctive and identifiable pattern for relevant and nonrelevant results (Li et al., 2018). Eye‐tracking data can show what search results, or what parts of documents, are attended to and use this as feedback for query expansion, refinement (Buscher et al., 2012), and even construction (Ajanki et al., 2009). Relevance, in the sense of binary text mining accuracy, can be detected from eye‐tracking with an accuracy of 64% (Liu et al. 2014), 74% (Gwizdka 2014), 80% (Bhattacharya et al. 2020), and 86% (Gwizdka et al. 2017).

The performance on predicting (binary) relevance decisions from eye‐tracking data confirms its potential for implicit feedback. Notwithstanding, the intuitive concept of relevance packs a vast complexity of human judgment and experience. Users apply a range of criteria when judging relevance, such as about the topicality, credibility, style, and reading level of a document (Schamber, 1994) and the story and visual effects of a movie (Adomavicius & Kwon, 2015). This vast complexity of human judgment subsequently shapes a cognitive‐affective experience of relevance (Ruthven, 2021). During an evolving interaction session, a particular set of metacognitive judgments and experiences unfolds, such as the case with users' reflection on processing dynamics: While cognitive ease is typically associated with feelings of satisfaction (Al‐Maskari & Sanderson, 2010), intermediate complexity seems associated with feelings of interest (Dubey & Griffiths, 2020; van der Sluis et al., 2014). The importance of these cognitive‐affective judgments and experiences during interaction indicate a potential for feedback “*beyond the conventional accuracy metrics*” (McNee et al., 2006, p. 1097).

Eye‐tracking offers a unique potential to unpack part of the complexity inherent to relevance and relevance decisions. Starting with the early work of Hess and Polt (1960, 1964), the eyes are known to reflect aspects of both cognitive processing and interest value. Subsequent research highlights that the eyes are particularly adept to reflect moment‐to‐moment cognitive processes (Just & Carpenter, 1980; Miller, 2015; Rayner, 1998). Among others, the eyes rest longer on words or regions that are difficult to process (Rayner et al., 2006) as well as attend to stimuli that offer intermediate levels of uncertainty in information theoretic terms (Gottlieb, 2012; Kidd & Hayden, 2015). It is thought that interested individuals direct attention and employ cognitive resources to maintain intermediate rates of information acquisition (Blain & Sharot, 2021; Graf & Landwehr, 2015; Kidd & Hayden, 2015). Their ability to offer continuous feedback makes it likely that tracking the eyes can reveal both the processing dynamics and attention patterns that are characteristic of interested individuals.

Notwithstanding the identified uses for and potential of eye‐tracking data for implicit feedback, it is unclear whether or not the unique potential of the eyes is as an indicator of (binary) relevance decisions, cognitive processing, or interest, nor how well these constructs can be distinguished using eye‐tracking data. Similar to the inherent ambiguity in behavioral traces data (Van der Sluis et al., 2017) and physiological signals (van den Broek, 2011), eye‐tracking data are difficult to interpret. Whether these cognitive processes become apparent through observing eye behaviors during reading is unclear, as many cognitive processes intertwine and observed effects are typically small and indistinctive when combined across word, sentence, and discourse levels (Rayner et al., 2006). In addition, eye‐tracking data are inherently noisy, in particular in ecologically valid settings. There is likely to be a significant variation in head position and distance, lighting conditions, and a low sampling frequency, especially with inexpensive consumer‐market eye trackers. Given these challenges, eye‐tracking feedback on cognitive‐affective processes is typically proposed and explored with highly controlled setups and stimuli (Rayner et al., 2006), which raises questions on its feasibility in an applied setting like text mining.

Incorporating both the opportunities and challenges that the eyes offer for implicit feedback, this paper presents a study that examines whether or not tracking the eyes can offer feedback above and beyond conventional accuracy metrics. We explore how well comprehensibility and interest can be identified and distinguished and we discuss how these aspects possibly feed back to inform text mining techniques. We frame interest following the emotion‐appraisal theory of interest, which considers interest as the momentary feeling‐of‐interest induced by an external stimulus, here texts (Silvia, 2006). This framing aligns with conceptualizations on situational interest but contrasts with more persistent personal or individual interests (Shin & Kim, 2019; Sinnamon et al., 2021). It furthermore hypothesizes that a certain level of processing difficulty is conducive to interest, but within limits of comprehensibility (Sinnamon et al., 2021; van der Sluis et al., 2014).

By exploring the ability of the eyes to unveil comprehensibility and interest, this study aims to bring a classical and long‐standing difference in the interpretation of eye behaviors, between cognitive activity (Hess & Polt, 1964) and interest value (Hess & Polt, 1960), within the context of human–information interaction. Next, we discuss the interpretation of eye‐tracking data. Subsequently, in section 3, the experimental method is described, including subsection 3.4. In section 4 the results are presented. Finally, in section 5 we discusses the results and reflect on the implications.

## 2 BACKGROUND: EYE BEHAVIOR

Our eyes follow a distinctive and identifiable pattern while reading. The task at hand (e.g., reading) and context (e.g., text) influence the major characteristics of eye behavior (Luke & Henderson, 2013; Rothkopf et al., 2007). Even though this means that eye behaviors are mostly determined by the task and text, higher‐order cognitive processes—like comprehension and interest—can change specific features of these behaviors (Luke & Henderson, 2013). Hess and Poll early on identified this potential of the eyes to reflect higher‐order mental processes: the interest in visual information (1960) and mental activity caused by problem solving difficulty (1964). This distinction reflects (a) the cognitive control hypothesis (or: eye‐mind link), which states that “the eye remains fixated on a word as long as the word is being processed” (Just & Carpenter, 1980, p. 330), relating fixations to cognitive processing, and; (b) the “like more, look more” assumption (or: information value link), which states that interested observers shift their gaze towards stimuli they value (Gottlieb, 2012; Miller, 2015). In the following subsections, we will review both hypotheses with a special attention for higher‐order cognitive processes.

### 2.1 The eye‐mind link

In self‐paced reading and eye‐movement research, the speed of reading and the movements of the eye are interpreted as indirect measurements of cognitive activity. During reading, the eyes make a series of rapid movements (i.e., saccades) separated by periods when the eyes are relatively still (called fixations). During the fixations new visual information is encoded from text. Fixations typically last about 200–250 ms, but neither shorter nor longer fixations are uncommon. Saccades typically last about 20–40 ms, depending on the distance traveled (Rayner et al., 2006). Saccades that move backwards (called regressions) form about 10% of all saccades in skilled readers and often travel short distances (i.e., one word). In comparison to eye movements, pupil size and blinks respond with a delay of 1.3 s to changes in human cognitive activity (Just et al., 2003), while they primarily respond to changes in brightness (Beatty & Lucero‐Wagoner, 2000).

Following the eye‐mind link, lower‐order processes related to word decoding and recognition are causally related to eye movements during reading. For example, the printed word frequency determines as much as 30–90 ms of total fixation duration per word (Inhoff & Rayner, 1986). Furthermore, readers perceive a word including and within its context. More semantically related words require less fixation time to process. Similarly, shorter and more probable words (cf., *n*‐grams) are more likely to be skipped over, increasing saccade length and shortening fixation times (Ehrlich & Rayner, 1981). A spill‐over effect can also be observed, when readers need to resolve an ambiguous meaning of a word. Word sense ambiguity increases fixation times, possibly delayed to subsequent words when the reader tries to get extra information before committing to a meaning, and can cause regressions, when the reader initially committed to a wrong meaning (Rayner & Duffy, 1986). These strong effects not only indicate that lower‐order processes related to lexical processing are causally linked to eye behaviors, but also that they can be modeled fairly accurately: Objective word and word‐context characteristics explain the major part of variance in aggregated number of fixations, fixation time, and saccade length (Rayner et al., 2006).

In understanding text, readers must be able to integrate information within and across sentences to form a coherent discourse representation. Overall, such higher‐order processes manifest itself through longer and more fixations, more time to read, and more regressions (Rayner et al., 2006; Schotter et al., 2014), while the sustained effort involved in comprehension is likely to increase pupil size (Just et al., 2003) and decrease its variability (Toker & Conati, 2017). For example, readers slow down at the end of sentences and subsequently show a larger saccade into the next sentence, as presumably readers wrap‐up the information in a sentence (Rayner et al., 2006). Also, fixations and regressions increase when readers encounter inconsistencies between sentences and look longer at pronouns, anaphora, and noun phrases when the antecedent is distant or difficult to identify. Contrary to lower‐order processes, however, higher‐order processes are not causally linked to eye behavior. They mainly become apparent when “*something doesn't compute*” (Rayner et al., 2006, p. 244), which is less common for skilled readers and for normal, well‐written texts. This leaves it unclear whether the highly controlled findings typical to reading comprehension studies extrapolate to models of comprehensibility in an applied context.

### 2.2 The information value link

Eye behavior is closely linked to interest. Overt visual attention is thought to share with interest that both seek to maximize information value (Gottlieb, 2012). Given a known task, observers shift gaze to sources that optimally reduce their uncertainty pertaining to the next appropriate action (Ballard & Hayhoe, 2009; Rothkopf et al., 2007). Interested observers—not driven by a task—similarly shift their attention to sources they value; as aptly captured by the “like more, look more” assumption. Interest value is associated with sources that offer a potential for uncertainty reduction and/or knowledge acquisition (Gottlieb et al., 2013; Graf & Landwehr, 2015; Silvia, 2006). The information value perspective of eye‐movement control draws close parallels between interest and eye movements. It casts eye movements in probabilistic, information theoretic terms optimizing some form of information value (Gottlieb, 2012; Gottlieb et al., 2013) and explains why eye movements, for example related to the relevance of search results, are fairly predictable (Buscher et al., 2012). This type of eye movement control depends on a known context where observers (and, similarly, experimenters) can estimate the expected information value of sources they can attend, for example by reading an abstract or by skimming headings.

Even though the information value perspective suggests a strong, formal, and granular link between interest and eye movements, the subjective experience of interest is generally considered a higher‐order construct that spans over longer periods of time than single gaze shifts (Silvia, 2008b). To a certain extent, such subjective experiences of interest are characterized by identifiable patterns in reading behavior as well (O'Brien et al., 2020). Readers spend more time on interesting sentences than on less interesting sentences (Wade et al., 1993), while interest tends to decrease rather than increase reading time at a discourse level (Shirey & Reynolds, 1988; van der Sluis et al., 2016). Once interested, readers presumably employ their mental resources more effectively and experience fewer distractions (Miller, 2015; van der Sluis et al., 2016). These findings suggest that distinctive eye behaviors both precede and follow on the activation of motivational resources typical to the experience of interest (Silvia, 2006). The distinctive patterns of interested readers might be detectable; for example, as differences between focal and ambient attention (Krejtz et al., 2016).

Promising examples exist of modeling higher‐order affective constructs related to interest. Jaques et al. (2014) predicted students' curiosity with 73.17% accuracy by modeling gaze transitions between various interface elements of an intelligent tutoring system and over fairly long episodes of interaction (14 min). Sims and Conati (2020) predicted users' confusion while using a visualization tool with up to 82% accuracy using a neural network trained with eye‐tracking features. Bixler and D'Mello (2016) predicted mind wandering while reading with up to 72% accuracy (chance level of 60%) using various classifiers with eye‐tracking features and text characteristics (i.e., difficulty). The differences in tasks, context, and constructs make it unclear whether these results replicate for interest during reading.

### 2.3 The challenge of co‐existence

As the preceding review shows, the influence of lower‐order processes via the eye‐mind link is well established, while the information value link interpretation is strengthening. These links support the understanding that eye behaviors are fairly predictable within a well specified task and context (Albrengues et al., 2019; Anderson et al., 2004; Chandra et al., 2020; Luke & Henderson, 2013). It indicates that the eyes are primarily driven by lower‐order processes; they “*do not do anything at the request of higher‐order cognitive processes that they would not do anyway*” (Luke & Henderson, 2013, p. 1241). In parallel, both cognitive (e.g., comprehension) and affective (e.g., interest) processing seems to influence specific features and patterns of eye behavior. Higher‐order processes are, however, not causally linked to eye behavior. This gives rise to a challenge of co‐existence—of different higher and lower‐order processes that influence eye behaviors through possible interactive and synergetic relationships (Goettker & Gegenfurtner, 2021; Kaakinen, 2021).

As already indicated by Hess and Polt's (1960, 1964) original claims, eye behaviors can be interpreted as both cognitive activity and interest. This seemingly co‐existence is explained by a theoretically intricate relationship between cognitive processing dynamics and the experience of interest (Graf & Landwehr, 2015; Silvia, 2006). To invoke interest, a text must contain a certain level of novelty and complexity, yet still remain comprehensible to a reader (O'Brien & McKay, 2016; Silvia, 2006; van der Sluis et al., 2014). A model of interest will therefore need to disentangle such distinctive processing patterns from the processing dynamics shared with comprehensibility.

Hess and Polt's (1960, 1964) original claims underpin the eyes' potential to disclose both readers' comprehensibility and interest. Co‐existence, however, poses a clear challenge to modeling higher‐order constructs using eye‐tracking. We decompose this challenge as follows:

1. How well can differences in comprehensibility be detected at discourse level for regular, nonexperimental (news) content?
2. Is it possible to unveil the experience of interest during reading?
3. Can we untangle the different interpretations of eye behaviors?

This triplet needs to be tackled to enable eye‐tracking‐induced individual feedback on both comprehensibility and interest during reading. In addition, reading is a learned behavior which accordingly shows substantial inter‐personal differences in eye behaviors (Carter & Luke, 2018; Payne et al., 2020). Similar to our iris and retina, also our eye behavior is personalized and, possibly, has biometric properties (van den Broek, 2010), which need to be accounted for. The next section will present the research methodology we use to address these challenges.

## 3 METHOD

Initial analyses on the subjective data are reported in van der Sluis et al. (2014). The current study reports on the potential of eye‐tracking to reveal comprehensibility and interest.

### 3.1 Participants and materials

Thirty volunteers (22 male, 8 female) with an average age of 28.60 (*SD* = 6.06) participated. None of them were native English speakers; but, all graded their reading literacy as high (*M* = 4.63; *SD* = .62; range 1–5), and all pursued or already obtained a university degree.

Eighteen articles from *The Guardian* ^1^ were sampled from a corpus of 14,856 articles at three levels of complexity: low, average, and high. Textual complexity was estimated using a computational analysis specified in van der Sluis et al. (2014). The analysis included seven textual features, including common features such as word frequency and novel features such as character and word‐level entropy.

**FIGURE 1:**
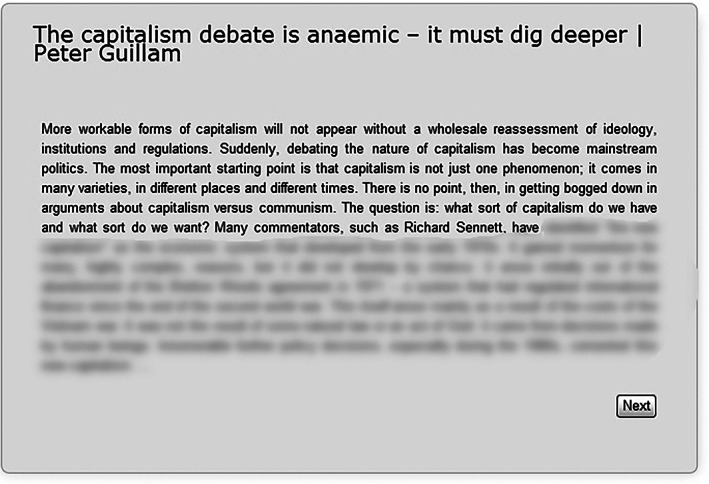
Example of article presentation. Content courtesy of Guardian News & Media Ltd. The article is partially blurred to comply with their open license terms

All articles were truncated after 1,200 characters. Three dots were added to indicate the story would normally continue. Any lay‐out was stripped from the articles, leaving only the title and textual content. The layout was not specifically designed for eye tracking. Rather it was intended to mimic (bootstrapped) designs common on the Internet. An example stimulus is shown in Figure 1.

### 3.2 Instruments

After each article was read, a questionnaire was administered to measure the following appraisals:

- *Comprehensibility* was measured by three 7‐point differentials, in accordance with related studies (e.g., Fayn et al., 2015; Silvia, 2008a): comprehensible–incomprehensible, coherent–incoherent, and easy to understand–hard to understand, which gave a high Cronbach's *α* = .893, *N* = 540 (Cronbach, 1951).
- *Interest* was measured using three items commonly used in interest studies (Silvia, 2005, 2008a, 2010). Two 7‐point differentials: interesting–uninteresting and boring–exciting and one 7‐point Likert scale asking participants to agree with the statement “I would be interested in reading more of this text.” The three items formed a consistent scale, confirmed by an excellent Cronbach's alpha of .921 (*N* = 540).
- *Novelty* and *complexity* were additionally measured but will not be analyzed in relation to eye‐tracking. These measures consisted of the following 7‐point semantic‐differentials: complex–simple (Silvia, 2008a), familiar–unfamiliar (Silvia, 2008a), and easy to read–difficult to read (Song & Schwarz, 2008).

These scales are commonly applied in experiments on the appraisal structure of interest for both textual stimuli and artwork (Fayn et al., 2015; Silvia, 2008a, 2010). Structural equation models confirmed that each of the scales covered a distinctive factor and that each explained a significant portion of variance in interest responses (see van der Sluis et al., 2014). The scales are furthermore shown to respond to theoretically related scales and manipulations, including coping potential and individual differences (Fayn et al., 2015; Noordewier & van Dijk, 2016).

### 3.3 Design and procedure

The experiment used a within‐subjects design that showed every article to every participant. For each participant, the articles were grouped in three counter‐balanced blocks based on their average topical familiarity scores. Within each block, the articles were shown in randomized order. The design controlled for the influence of novelty in order to isolate the influence of articles varying in complexity on comprehensibility and interest.

The experiment started with instructions and an initial questionnaire on basic demographics and topical familiarity. Participants were told that the experiment queried their interest in different news articles and that reading was self‐paced. Each block started with textual instructions and ended with the closing questionnaire. Each of the 18 articles were followed by the above mentioned scales. The full experiment lasted around 45 min. Some participants indicated this demanded a lot of their concentration. The eye‐tracker was calibrated before the start of the experiment. The experiment took place in a dedicated room with only artificial and thereby controlled lighting.

### 3.4 Eye‐tracking apparatus and analysis

**FIGURE 2:**
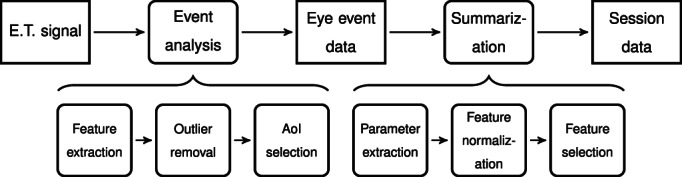
The processing pipeline applied to the eye‐tracking (E.T.) signal

Here we describe the eye movement acquisition, as shown in Figure 2. We used a SMI RED60 binocular eye‐tracker at 60 Hz and BeGaze 3.0.181 software to track the participant's gaze on a standard 22″ TFT monitor (resolution: 1280 × 1024). One participant's eye‐tracking data was lost due to a software failure.

The forthcoming feature (and *parameter*) set contains features regularly used in reading studies, complemented with saccade and regression speed to capture differences in reading speed as generally seen with interested readers:

- Fixations (*count*, *duration*) were detected using a dispersion‐based algorithm (Blignaut, 2009), with a pre‐specified minimum duration of 80 ms.
- Saccades (*count*, *amplitude*, *duration*, *peak speed*): the connection between two subsequent fixations. The eye‐tracker registered blinks as saccades with a 0, 0px position, which were excluded from further analysis.
- Regressions (*count*, *amplitude*, *duration*, *peak speed*): saccades with a northwards (*y*‐axis) and horizontal (*x*‐axis) direction of minimal 20px distance. This overlaps approximately with the size of the word “the” on the screen and excludes regular, progressive saccades as well as microsaccades that presumably correct displacements in eye position.
- *Coefficient K* (Krejtz et al., 2016, p. 4):
(1) $$K=\frac{1}{i}\sum_{i}\left(\frac{d_{i}-\mu_{d}}{\sigma_{d}}-\frac{a_{i-1}-\mu_{a}}{\sigma_{a}}\right),$$
  where *a*_*i*+1_ is the saccade amplitude, *d*_*i*_ the *i*th fixation duration and *μ*_*d*_, *σ*_*d*_, *μ*_*a*_, and *σ*_*a*_ are the participant's mean and standard deviation of the fixation duration and saccade amplitude (see p. 15 on normalization). This measures the ratio between focal attention, characterized by longer fixations indicative of a central mode of visual processing, and ambient attention, characterized by longer saccades indicative of an exploratory mode of processing (Krejtz et al., 2016).

- *Pupil size* (in pixels): the normalized (see Equation 2) average pupil diameter across both eyes during a fixation. Because of varying baseline levels in pupil size across people, non‐normalized pupil size was not included.
- *Reading time* (in seconds): the duration between the first and last fixation per article.

For each feature, the interquartile range (iqr) was computed. Data points laying outside a limit of 4 times the iqr below the second or above the third quartile were identified as outlier and removed. This is a rather lenient limit (e.g., saccades of several seconds) and allows for rather skewed distributions (e.g., saccade duration). This resulted in a total of 285,190 fixations, 259,286 saccades, 34,605 regressions, and 196,613 *K* samples, a reduction of, respectively, 0.50, 15.35, 6.80, and 1.59%.

Eye events not in the area of interest (i.e., the article title or content) were removed. This resulted in 168,914 fixations, 154,560 saccades, 18,450 regressions, and 88,723 *K* samples.

The eye event data were summarized per reading session. Subsequently, for each feature, the statistical parameters count, mean, variance, skewness and kurtosis were calculated to represent the signal's distribution. The statistical moments variance, skewness, and kurtosis were included to capture both discourse‐level and local effects on reading patterns (see section 2).

To tackle inter‐ and intra‐personal differences in eye behavior, all data were normalized as follows (van den Broek, 2011, p. 87):

(2) $$\tilde{f}\left(t\right)=f\left(t\right)-\mu ,$$

with *μ* being participant's personal baseline from the original feature series *f*(*t*). The normalization was executed twice with *μ* being respectively participant's median during the experiment (annotated with ^*a*^) and the respective experimental block (annotated with ^*b*^). Coefficient *K* was an exception on this, as it was already normalized (see Equation 1). Such a normalization step is standard in psychophysiological and oculometric studies and is both suitable and reliable for absolute level comparisons (van den Broek, 2011). As each of these normalizations will likely (un)cover different sources of variability they will each be included to the subsequent feature selection and statistical modeling steps. The resulting data set contained in total 130 variables and 522 observations.

To secure further processing, checks were executed for missing values, intra‐variable variance, and between‐variable correlation. In total, five observations were removed that contained missing values, none of the variables had near‐zero variance, and five variables were highly correlated (*r* > .95) and subsequently removed: saccade count, *K* skewness^*a*^, and *K* kurtosis^*b*^. The final data set contained 127 variables for 517 reading episodes.

### 3.5 Statistical analysis

**FIGURE 3:**
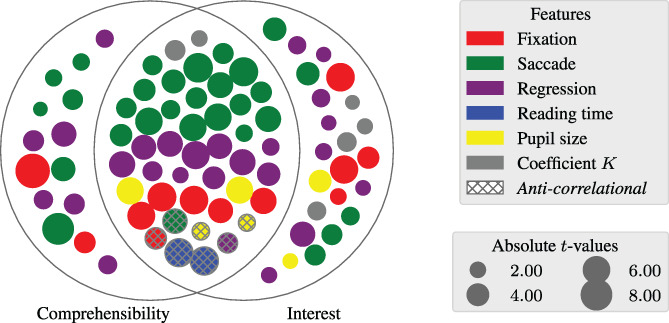
Importance of predictors for the interest and comprehensibility regression models. Absolute *t*‐values are averaged for shared predictors. Anti‐correlational refers to predictors with opposing signs for both models

**FIGURE 4:**
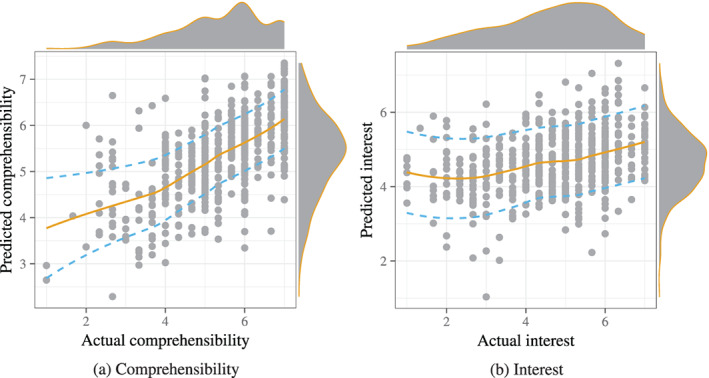
Predictive performance of statistical models for comprehensibility (a) and interest (b) in a leave‐one‐out cross‐validation setting. The scatterplots show the actual and predicted values, accompanied by a trend line and 95% confidence intervals for the predictions. Above and to the right of the scatter plots, density plots illustrate the distribution for the shown values

Statistical analyses were performed in R using packages *outliers*, *caret*, *Hmisc*, *MASS*, *ggplot2*, and *ggextra*. A regression analysis was performed in two steps. Firstly, for both comprehensibility and interest, a linear regression model (LRM) was trained on the session data with stepwise variable reduction. The explanatory performance of the regression models was evaluated using *R* ^2^. The overlap between the resulting predictor sets (after variable reduction) is illustrated in Figure 3. Second, the reduced set of predictor variables from the linear models was used to predict unseen observations in a leave‐one‐out cross‐validation setting. For each observation out of *N* total observations, a LRM was trained (without variable reduction) on *N* − 1 observations and tested on one observation. The set of predictors was thus assumed to be an optimal set, while the coefficients were retrained with every evaluation pass. The predictive performance was evaluated using min−max normalized root‐mean‐square error (nRMSE) and Pearson's correlation *r* (see Figure 4). Figure 4 shows density plots and a trend line with 95% confidence intervals, calculated using locally weighted smoothing with R's *loess* and *predict.lm* methods.

## 4 RESULTS

### 4.1 Descriptive statistics

Participants appraised the 18 articles as comprehensible (*M* = 5.35, *SD* = 1.23) and interesting (*M* = 4.66, *SD* = 1.43) (scale: 1–7). Comprehensibility and interest appraisals share 21.69% of variance. Table 1 gives the eye‐tracking features and their parameters' averages. Two of Table 1's values differ from typical values (see section 2). The mean fixation length was 367.35 ms, *SD* = 112.27), where a typical fixation takes 200–250 ms. Regression duration was longer than mean saccade duration with a mean of 71.08 ms (*SD* = 32.45), this can however be expected as microsaccades were excluded by the specification of regressions (see Section 3.4).

**TABLE 1:** Average values of the eye‐tracking variables per article reading session

Feature	Sum	Mean	Var	Skewness	Kurtosis
Reading time (s)	75.01				
Fixation					
Count	324.83				
Duration (ms)		367.35	55.83	1311.96	2536.82
Pupil size (px)			0.79	−0.05	5.46
Regression					
Count	35.62				
Amplitude (°)		16.41	318.84	1.37	1.75
Duration (ms)		71.08	4.31	1198.80	1089.58
Peak speed (°/s)		851.99	283,408.86	0.31	−0.66
Saccade					
Count	297.80				
Amplitude (°)		2.67	19.46	2.66	9.02
Duration (ms)		28.96	0.60	2460.64	6823.96
Peak speed (°/s)		179.40	57,916.98	2.60	9.13
Coeff. *κ*		0.04	0.67	−0.15	1.94

Table 2 gives the significant correlations between eye‐tracking variables and comprehensibility and interest. The correlational analysis shows strong effects for reading time, which correlated with both comprehensibility and interest both before and after normalization (^*a*,*b*^): Easier to comprehend texts need less time to read and interested readers read faster. This is also reflected in the counts of fixations, saccades, and regressions. These all decrease significantly when less time is spent reading.

**TABLE 2:** Correlations (*r*) between eye‐tracking variables and comprehensibility (comp.) and interest (inte.) appraisals

Variable	Correlation (*r*)		Variable	Correlation (*r*)		Variable	Correlation (*r*)	
	Comp.	Inte.		Comp.	Inte.		Comp.	Inte.
rea. tim.	−.144*	−.124**	reg. amp. avg.	−.089***		sac. dur. var.^*b*^	−.090***	−.091***
rea. tim.^*a*^	−.226*	−.160*	reg. spe. ske.		−.129**	sac. dur. ske.		.090***
rea. tim.^*b*^	−.225*	−.164*	reg. spe. kur.		−.102***	sac. dur. ske.^*b*^		.112***
pup. avg.^*a*^	−.113***		sac. amp. avg.^*a*^	−.117**		sac. spe. avg.^*a*^	−.120**	
pup. var.	−.177*	−.092***	sac. amp. avg.^*b*^	−.137**	−.091*	sac. spe. avg.^*b*^	−.124**	
pup. var.^*a*^		−.088***	sac. dur. avg.		−.095***	sac. spe. kur.	−.099***	
pup. kur.	.086***		sac. dur. avg.^*b*^	−.104***	−.125**	*κ*. var.^*a*^		−.088***
fix. dur. var.^*a*^	−.088***	−.094***	sac. dur. var.		−.103***			

*Note*: Variables annotated with ^*a*^ and ^*b*^ are normalized per participant and per participant‐experimental block, respectively (see Equation 2).

Abbreviations: rea. tim., reading time; fix., for fixation; reg., regression; sac., saccade; dur., duration; pup., pupil; amp., amplitude; spe., peak speed; avg., average; var., variance; ske., skewness; kur., kurtosis.

* *p* < .001; **p < .01; ***p < .05.

Average pupil size correlated with comprehensibility, indicating less effort was exerted, while its variance decreased with both comprehensibility and interest. For normalized fixation duration, its variance similarly decreased with comprehensibility and interest. This suggests a more fluent and consistent reading behavior with both comprehensibility and interest.

For saccades, their amplitude, duration, and speed had several significant relationships. The overall picture of these relationships is that, with comprehensibility, saccades were shorter, took less time, and had a lower peak speed. For interest, saccades showed similar relationships with the exception of peak speed. Interested readers furthermore showed less variance and higher skewness in the distribution of their saccade duration. Regressions showed two effects: their amplitude decreased with comprehensibility whereas the skewness and kurtosis of peak speed increased with comprehensibility, suggesting distinctive distributions of regressions between comprehensibility and interest. In addition to saccades and regressions, normalized Coefficient *K* variance decreased with interest, indicating less variation between focal and ambient modes of visual processing.

### 4.2 Linear regression

The LRM for comprehensibility consists of 60 parameters, *R* ^2^ = 49.93, *F*(59, 454) = 7.67, *p* < .001. The LRM for interest consists of 69 parameters, *R* ^2^ = 30.41, *F*(68, 445) = 2.86, *p* < .001. Stepwise variable reduction was used to reduce the LRM's dimensionality.

Figure 3 illustrates the number of unique and shared predictors for both models, including whether or not the direction of correlation is shared. The model for comprehensibility contains 14 unique predictors. The model for interest contains 23 unique predictors. In total 45 features are shared between the models, of which 38 shared the same sign and 7 have opposing signs. Figure 3 furthermore shows that fixation, saccade, and regression predictors are present in both models as well as in their overlap.

Noteworthy from Figure 3 are the influences of reading time, pupil size, and Coefficient *K*. Reading time has a strong, yet opposing influence on both models which offsets the consistently negative correlation between reading time and interest and comprehensibility (see Table 1). Pupil size has four out of six features predictive of interest or anti‐correlational, which contrasts with the typical relation found between pupil size and cognitive effort. Finally, Coefficient *K* is mostly related to the interest model with four out of six predictors exclusive to it. These observations indicate that these three features can help discriminate between comprehensibility and interest when considered in co‐dependence with other included predictors.

To assess the predictive performance of both LRMs, we applied leave‐one‐out cross‐validation. The LRMs predicted *r* = .608 and *r* = .329 of comprehensibility and interest ratings, with normalized errors of respectively nRMSE = .164 and nRMSE = .232. Figure 4 illustrates this predictive performance. It relates the model predictions to the actual appraisals of participants, including the 95% confidence intervals for the predictions. Furthermore, the figure shows the distributions of actual and predicted values.

Figure 4a shows a possible detriment to predictive performance. Due to a lack of training data, the comprehensibility model is less reliable at low levels of comprehensibility. At medium and high comprehensibility, the relation between predicted and actual values is close to an optimal diagonal line, accompanied by small confidence intervals. Figure 4b shows a low predictive performance for interest. This is further shown by the difference in data distributions between the predicted and participants' appraised interest.

## 5 DISCUSSION

We shed light on a classic, over half a century standing discrepancy in the interpretation of eye behaviors: processing (Hess & Polt, 1964) versus value (Hess & Polt, 1960). Eye‐tracking data enabled to delineate aspects of cognitive processing and, to a lesser extent, interest value. The LRMs explained 49.93 and 30.41% variance (*p* < .001) for respectively comprehensibility and interest, with a predictive performance of *r* = .608 and *r* = .329, respectively. These findings confirm the eyes' potential to offer feedback above and beyond conventional accuracy metrics.

This study is the first to benchmark and compare comprehensibility and interest in an eye‐tracking study. Other studies did evaluate the ability of eye‐tracking to predict related constructs, such as relevance judgments with 64–86% (Gwizdka et al., 2017; Liu et al., 2014), mind wandering during reading with 72% (12% above chance level) (Bixler & D'Mello, 2016), curiosity with 73% (Jaques et al., 2014), and confusion with 82% accuracy (Sims & Conati, 2020). However, there are notable differences with the approach taken in the present study. These studies evaluated a binary classification accuracy rather than a continuous regression problem. Baseline chance levels are much lower for a 7‐point (14.29%) than a binary scale (50.00%) (van den Broek et al., 2013). Moreover, typically auxiliary features about the text and user‐interface interaction were added as predictors while longer time frames were recorded. By focusing exclusively on short reading sessions, we addressed a notably hard problem. Further, by using only event‐based eye‐tracking features and straightforward regression techniques, the present work shifted focus away from machine learning techniques towards the value and possibilities of the features and their interpretation in terms of the predicted constructs (Rudin, 2019). Taken together, exploring a long standing discrepancy against a 14.29% baseline, the 49.93% (comprehensibility) and 30.41% (interest) explained variance can be regarded a promising performance.

The findings support the proposition that appraised comprehensibility can be detected from the eyes. The text passages used were all well‐written news articles, regarded as fairly comprehensible (*M* = 5.35, *SD* = 1.43). This shows that small differences in comprehensibility can already be detected from the eyes. This is further supported by the small confidence intervals and nearly linear fit between predicted and appraised comprehensibility (see Figure 4a). This result is distinct from earlier findings as typically word‐level difficulties average out at a discourse level and discourse‐level difficulties only become visible when large, often artificial obstacles are encountered (Rayner et al., 2006). The result indicates that higher‐order processes related to comprehension, even though not causally linked to eye behavior, can nonetheless be detected from the eyes. It remains a question however whether our model generalizes across other genres of texts or whether effective models can only be learned for specific genres of text.

In line with Hess and Polt (1960, 1964), the current findings show that the eyes not only reveal aspects of cognitive activity but also of interest. Our results particularly confirm original findings that pupil size variations have distinctive distributions for comprehensibility and interest. The predictive performance of the interest model nevertheless also underlines the challenge of detecting interest from the eyes. Using eye‐tracking data, it seems hard to distinguish comprehensibility from interest. Both the comprehensibility and interest models share the majority of predictors (45 variables). A likely interpretation for this overlap comes from the theoretical importance of comprehensibility as key appraisal for interest (Silvia, 2006). In particular, the overlap between predictors points to the intervening role of complexity: Textual complexity can *increase* interest by stimulating the reader while at the same time *decrease* interest by reducing comprehensibility (van der Sluis et al., 2014). This indicates that distinctive processing dynamics—complex yet comprehensible—likely underlie the experience of interest (Graf & Landwehr, 2015; Silvia, 2006) as well as explains the overlap of predictors between comprehensibility and interest.

An alternative interpretation for the overlap between predictors comes from possible psychometric and oculometric limitations. From a psychometric perspective, users' introspective judgments are overall influenced by positive affect and top‐down processing expectations. This puts a natural limit on the divergent validity of measures of comprehensibility and interest. A shared variance of 21.69% confirms some degree of overlap, as is also theoretically expected, but nevertheless indicates that the two constructs can be differentiated subjectively. From an oculometric perspective, the eyes do not readily reveal interest as suggested by the “like more, look more” assumption. Our confirmation of a negative correlation between reading time and interest rather indicates that interested readers employ their mental resources more effectively—with likely finegrained influences on their eye behavior. With 23 unique predictors for the interest model and 45 features shared with the comprehensibility model, the current results can neither confirm nor refute the possibility of disentangling interest from comprehensibility using eye‐tracking data. Our results rather indicate a need for specialized eye‐tracking features and subjective instruments that can identify and distinguish interest's distinctive processing dynamics. A case to the point is the ratio between focal and ambient attention as measured by coefficient *K* (Krejtz et al., 2016), which showed a distinctive distribution for interest.

The entanglement of higher‐order processes is inherent to the “co‐existence challenge”: Higher‐order processes combine to influence specific features of eye behavior, while lower‐order processes influence eye behavior's major characteristics. The deterministic nature of the eye‐mind link and information value link offers a partial solution to this co‐existence. The eye‐mind link causally relates fixations to the lexical processing of words whereas the information value link probabilistically relates gaze shifts to uncertainty reduction and knowledge acquisition. These links indicate that a large portion of variability in eye behavior stems from textual characteristics and relevance rather than from higher‐order effects of comprehensibility and interest. Modeling the characteristics of a text region (Bixler & D'Mello, 2016; van der Sluis et al., 2014) as well as the relevance of those regions can help interpret eye behaviors at word and discourse level. Such models have the potential to relate and distinguish higher‐order from lower‐order processes, including the relation between word processing and comprehensibility and between relevance and interest, and will likely improve predictions of comprehensibility and interest beyond the current results.

Through exploring implicit measures of comprehensibility and interest, this study aimed to unpack part of the vast complexity of human judgment and experience inherent to relevance. Of these measures, comprehensibility has traditionally been associated with the cognitive relevance or pertinence of information in relation to instrumental information needs. This relevance space is typically considered within task‐based situations in which (more or less) objective and intellectual criteria dominate relevance decisions (Saracevic, 2007; Xu, 2007). Interest is instead associated with interactions that are more hedonic and affective in nature (Ruthven, 2021; Xu, 2007). Even though a vast variety of interactions seem not driven by instrumental needs anymore, this emotional side of relevance has arguably been less well integrated into theorizing on relevance (Belkin, 2008; Ruthven, 2021). Our results rather highlight an intricate relationship between cognitive processing dynamics and the affective experience of interest. This conclusion is in line with the emotion‐appraisal theory of interest, which postulates that interest, and emotions in general, follow from (subliminal) cognitive appraisals (Ellsworth & Scherer, 2003; Silvia, 2008b). This suggests that the non‐instrumental, emotional side of relevance follows from what is commonly considered as cognitive relevance. Rather than being dichotomous, the cognitive and affective sides on relevance relate closely.

The presented study furthermore contributed to an understanding of the cognitive judgments that are key to users' affective experience as well as the extent to which they can be revealed for feedback. Knowing which judgments are central and can be revealed opens up for new forms of adaptation. The right types of feedback can explain why something was relevant, which potentially offers an improved understanding of users' information needs. It furthermore can turn users' cognitive‐affective experience with the retrieved information into an objective for information systems. This potentially offers various new forms of interaction support that help users cope with the complexities of information and knowing (e.g., Taranova & Braschler, 2021). These new opportunities for adaptation will be instrumental for information systems to enter into cooperative relationships with their users, where systems increasingly consider subjective aspects of information interaction until they eventually cater for information that challenges yet resonates with its users (Ruthven, 2021).

Even though the wide‐spread application of eye‐tracking as implicit feedback for text mining is unlikely to be realized in the near future, the present study indicated its potential usefulness. Tracking the eyes offers a unique potential to reveal processing dynamics that underlie both cognitive and affective aspects of information interaction. This conclusion is in line with the observation made by Hess and Polt (1960, 1964) over half a century ago: the eyes can reveal aspects of both cognitive activity and interest value. In particular, the current work shows that eye movements unveil discourse‐level comprehensibility and that particular cognitive processing dynamics partly underlie the experience of interest. The contribution of the proposed types of continuous observation provides an opportunity for new system design. For systems that deliver personalized information, optimized ‘beyond the conventional accuracy metrics’ for how we process and experience information.

## Endnote

^1^

www.guardian.co.uk
